# Lipopolysaccharide treatment suppresses spontaneously developing ankylosing enthesopathy in B10.BR male mice: The potential role of interleukin-10

**DOI:** 10.1186/1471-2474-13-110

**Published:** 2012-06-21

**Authors:** Jana Capkova, Tomas Hrncir, Alena Kubatova, Helena Tlaskalova-Hogenova

**Affiliations:** 1 Laboratory of Diagnostics for Reproductive Medicine, Institute of Biotechnology AS CR, v.v.i., Videnska 1083, Prague 142 20, Czech Republic; 2 Department of Immunology and Gnotobiology, Institute of Microbiology AS CR, v.v.i., Doly 183, Novy Hradek 549 22, Czech Republic

## Abstract

**Background:**

Ankylosing enthesopathy (ANKENT) is an animal model of human ankylosing spondylitis. ANKENT is an inflammatory disease affecting the ankle and tarsal joints of the hind limbs in susceptible mouse strains. In the disease, the participation of intestinal microbiota components was suggested. Therefore, we attempted to increase the incidence of ANKENT by systemic administration of lipopolysaccharide (LPS), which is a component of bacterial cellular walls and stimulates inflammatory processes.

**Methods:**

ANKENT occurrence, serum cytokine profiles, spleen cellular composition and *in vitro* cytokine response to LPS were analysed in LPS-treated and control LPS-untreated B10.BR male mice.

**Results:**

Contrary to expectations, LPS treatment decreased the incidence of ANKENT in LPS-treated group compared to control LPS-untreated group. Flow cytometry analysis of splenocytes showed an increased percentage of macrophages, dendritic cells and neutrophils and a decreased percentage of B cells, T cells and T helper cells in LPS-treated males following LPS administration. In addition, LPS-treated males had significantly elevated IL-6 and IL-10 serum levels. At 20–22 weeks after the final LPS application, splenocytes from LPS-treated mice were more susceptible to *in vitro* LPS stimulation than those of the controls and produced significantly higher levels of TNFα and IL-6.

**Conclusions:**

Repeated systemic stimulation with microbial component lipopolysaccharide in early adulthood significantly reduced the incidence of ANKENT in B10.BR mice and this finding can support the “hygiene hypothesis”. In LPS-treated mice, the innate immunity parameters and the level of anti-inflammatory IL-10 cytokine were significantly increased. Nevertheless, the immunological mechanism of the LPS protective effect remains unclear.

## Background

Ankylosing spondylitis (AS) is a serious rheumatological disease resulting in patient disability. The disease affects mainly men and the first symptoms usually manifest themselves in early adulthood. A relevant genetic risk factor for AS is the HLA-B27 allele: 96% of AS patients carry the HLA-B27 gene [1,2]. To study the causes of AS, various biological models were created [3-6].

Our animal model for AS shows disease of the ankle and tarsal joints in the hind paws of mice. The disease affects joints and entheses, which are the areas of insertion of ligaments, tendons or joint capsules into bone. Analogously to human spondyloarthropathies (SpA) [7,8], enthesitis is a specific marker of the affected joints and the disease was classified as **ank**ylosing **ent**hesopathy (ANKENT) [9]. The disease occurs spontaneously in some inbred mouse strains [10,11] and almost exclusively in males [12]. Its incidence is significantly increased in HLA-B27 mice [11], confirming an implication of the B27 allele in ANKENT development.

The disadvantage of the ANKENT model is the relative low frequency of the disease - about 10% B10.BR male mice under conventional (CV) and about 5% mice under specific pathogen free (SPF) conditions naturally develop ANKENT [11].

In previous studies, we found that germ-free mice remain ANKENT free [13] and their colonization with a mixture containing a low number of common intestinal bacteria (*Bacteroides sp., Enterococcus sp., Staphylococcus sp., Veillonella sp.)* alone results in ANKENT development [14]. Because mucosal barrier impairment and the consequent increased penetration of commensal microbiota components were suggested to play a role in the disease development [15], we attempted to induce ANKENT and increase its frequency through the systemic administration of lipopolysaccharide (LPS). LPS, a component of bacterial cellular walls, is an efficient stimulator of the immune inflammatory response [16] and its intraperitoneal application increases intestinal permeability [17]. A further aim of our work was to study the immunological profile of ANKENT-susceptible B10.BR mice during LPS administration and at the end of the observation period and to compare it to that of LPS-untreated control mice.

## Methods

### Mice

**Figure 1:**
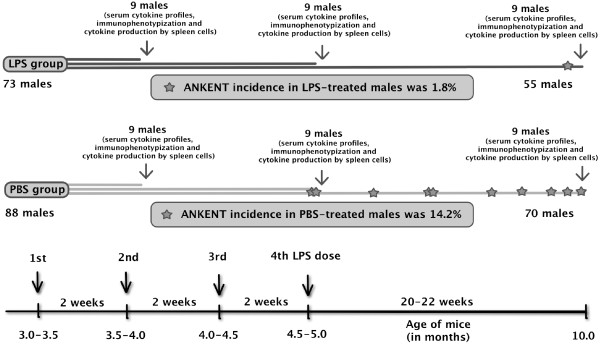
**ANKENT occurrence and LPS administrations.** LPS or PBS was administered i.p. to B10.BR males from the age of 3–3.5 months in two-weeks intervals (four times). The ANKENT occurrence was recorded throughout the whole experiment. The incidence of ANKENT was significantly lower in the LPS-treated group (1/55) compared to control PBS group (10/70; Fisher’s exact test p < 0.02). To analyze immune parameters sera and spleens were collected three days after the second and the fourth LPS dose and at the end of experiment (3x9 mice of each group).

ANKENT- susceptible inbred B10.BR (C57BL/10 genetic background, H-2^k^ haplotype) male mice born to females at the age of 2 ½ - 3 months were used in the experiments. The litters were split before sexual maturity and males from each litter were divided into two groups: LPS-treated and control LPS-untreated (PBS-treated) group. In the experiment there were 73 LPS-treated males and 88 LPS-untreated control males. In the course of LPS administration, after the second and the fourth LPS dose, and at the end of the experiment, *i.e.* 20–22 weeks after the fourth LPS dose, always 9 LPS-treated and 9 control males were sacrificed and sera and spleens collected (Figure 1). All mice were housed under the same conventional conditions, fed the same diet and caged 4–6 males together.

All experiments were approved by the Institutional Animal Care Committees of the Institute of Biotechnology and the Institute of Microbiology and were in accordance with the EC Directive 86/609 EEC for animal experiments.

### ANKENT screening and evaluation

ANKENT is a progressive stiffening of the ankle and tarsal joints in the hind paws of mice. Joint stiffness was evaluated by gentle stretching once a week and a score between 0 (normal mobility) and 3 (completely immobile) was assigned. Scores of 2 and above were regarded as clearly positive for ANKENT. Joints of some ANKENT positive animals were also checked by radiography. The mice were observed until they reached the age of 10 months.

### LPS and its administration

LPS was isolated from E. coli O83 and purified by phenol-water extraction [18]. Twenty mg of LPS was dissolved in 20 ml of sterile PBS and the solution was administered immediately or aliquoted and stored at – 20 °C until used. Mice were intraperitoneally (i.p.) injected with LPS (100 μg LPS/0.1 ml PBS per mouse) four times at two-week intervals. At the beginning of LPS treatment the males were 3-3^1^/_2_ months old. Control LPS-untreated mice were injected i.p. with 0.1 ml PBS according to the same schedule.

### Antibodies

The following monoclonal antibodies with matching isotype controls were used: PE-conjugated anti-mouse CD19 for detection of B cells; FITC-conjugated anti-mouse CD3e, FITC-conjugated anti-mouse CD4, PE-conjugated anti-mouse CD8a and PE-conjugated anti-mouse Foxp3 for detection of T cells and their subpopulations; PE-conjugated anti-mouse CD49b for detection of NK cells; PE-conjugated anti-mouse CD11b, FITC-conjugated anti-mouse CD11c, and PE-Cy5 conjugated anti-mouse Ly-6 G for detection of leukocyte populations. All monoclonal antibodies were purchased from eBioscience, USA.

### Flow cytometry

Phenotypic analysis of cells isolated from the spleen was performed using flow cytometry (FACS). Cells were resuspended in ice-cold FACS buffer (1% bovine serum albumin and 0.1% sodium azide in PBS) to a concentration of 2 x 10^7^ cells/ml and pre-incubated with 1 μg of anti-mouse CD16/CD32 per 10^6^ cells for 5 min on ice prior to staining. Antibodies were diluted to predetermined optimal concentrations in 50 μl of FACS buffer and dispensed into wells of a 96-well microtiter plate. Fifty μl of the cell suspension was added to each well and incubated for 20 min at 4 °C in darkness. Then, the cells were washed twice and resuspended in 100 μl of FACS buffer. Intracellular staining of mouse Foxp3 was performed using PE anti-mouse Foxp3 Staining Set according to the manufacturer’s protocol. Sample data were acquired using FACSCalibur flow cytometer (Becton Dickinson, USA) and analysed with FlowJo software (Tree Star, USA).

### Serum samples

Blood samples were collected the third day after the second and the fourth LPS dose and at the end of experiment (Figure 1). Not treated blood was let stand for 2 h at room temperature, centrifuged and sera were collected and stored at −20 °C until cytokine analysis.

### *In vitro* cytokine stimulation

Spleen cells were cultured at 2 x 10^6^ cells/ml in a 96-well flat-bottom culture plate in complete RPMI-1640 medium. The cells were stimulated with 10 μg/ml LPS and incubated in 5% CO_2_ at 37 °C for 48 h.

### Multiplex cytokine determination

Cytokine levels in sera and supernatants were detected using fluorescent beads coated with analyte-specific capture antibodies (BioSource, USA) according to the manufacturer’s protocols. The beads were analyzed using the Bio-Plex System (Bio-Rad Laboratories, Hercules, USA).

### Statistical analysis

Statistical differences in ANKENT occurrence between LPS-treated and control groups were evaluated by Fisher’s exact test (two-tailed). P values less than 0.05 were considered statistically significant.

Data concerning the proportional representation of cell populations and cytokine levels are expressed as mean ± standard deviation (SD). Statistical differences between LPS-treated and control groups were examined using the Student’s *t* test. A p-value of <0.05 was considered statistically significant. Statistical significance was analyzed using Prism 5.0 statistical software (GraphPad Software, San Diego, CA).

## Results

### ANKENT occurrence

**Figure 2:**
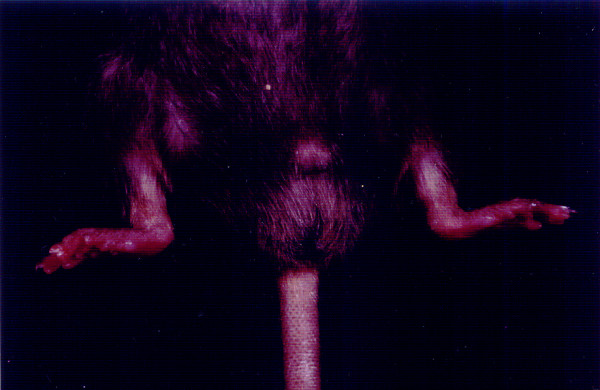
**ANKENT in both paws.** A 7-month-old ANKENT-positive male from the control LPS-untreated group with both paws affected.

**Figure 3:**
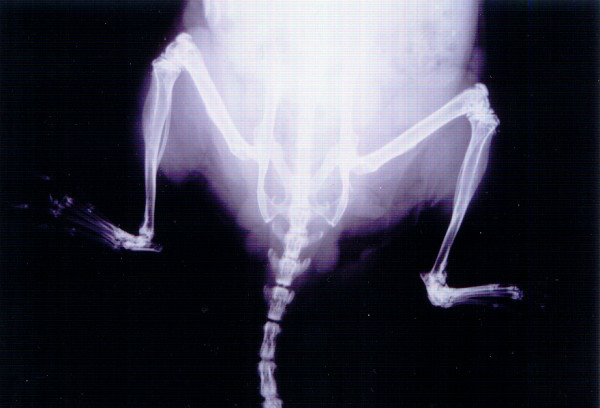
**A radiograph of ANKENT-positive male.** A control LPS-untreated male with the right paw affected. The left paw is normal.

ANKENT occurs particularly in inbred mouse strains with the C57BL genetic background. The disease begins with mild swelling and redness in the ankle area and in a short period of time (2–4 weeks) culminates in irreversible stiffening of the ankle and tarsal joints (Figure 2). Proliferative inflammation of the joints and adjacent tissues causes ossification of the joint cartilage [10] which is well seen on a radiograph (Figure 3).

Among LPS-treated males we found only one ANKENT positive male, *i.e.* 1.8% (1/55) incidence. On the other hand, ANKENT occurrence in the control LPS-untreated males was relatively high and joint disease developed in 14.2% (10/70) cases (Figure 1). Statistical evaluation by Fisher’s exact test demonstrated a significant difference in ANKENT incidence between LPS-treated and control males (p < 0.02).

### Leukocyte subpopulations in the spleen

**Figure 4:**
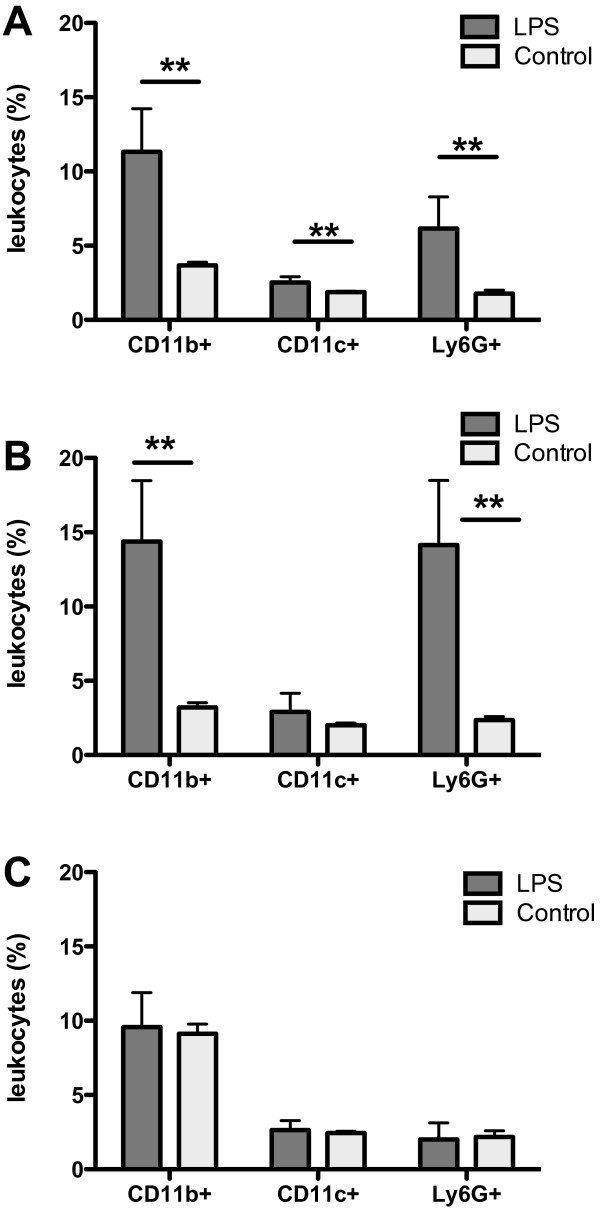
**FACS analysis of leukocyte subpopulations in the spleens of LPS-treated and control LPS-untreated B10.BR males.** After the second LPS dose the percentage of macrophages (CD11b+), dendritic cells (CD11c+) and neutrophils (Ly-6G+) was increased in LPS-treated males (**A**) and the increase of CD11b + and Ly-6G+ cells lasted after the fourth LPS dose (**B**). No significant differences in the percentage of CD11b+, CD11c + and Ly-G6+ cells were found between both groups 20–22 weeks after the fourth LPS dose (**C**). * p < 0.05, ** p < 0.01.

FACS analysis of spleen cells showed that LPS stimulation affected the proportion of macrophages (CD11b+), dendritic cells (CD11c+) and neutrophils (Ly-6G+) in the spleen (Figure 4). After the second LPS dose, LPS-treated males had a significantly higher percentage of CD11b+, CD11c + and Ly-6G+ leukocytes (p < 0.01) compared to control LPS-untreated males (Figure 4A). The increased percentage of CD11b+ and Ly-6G+ cells (p < 0.01) also persisted after the fourth LPS dose (Figure 4B). However, at the end of the experiment both LPS-treated and control males had almost identical proportions of leukocyte subpopulations (Figure 4C) and no statistical differences were found between the LPS-treated and the control group.

### Lymphocyte subpopulations in the spleen

**Figure 5:**
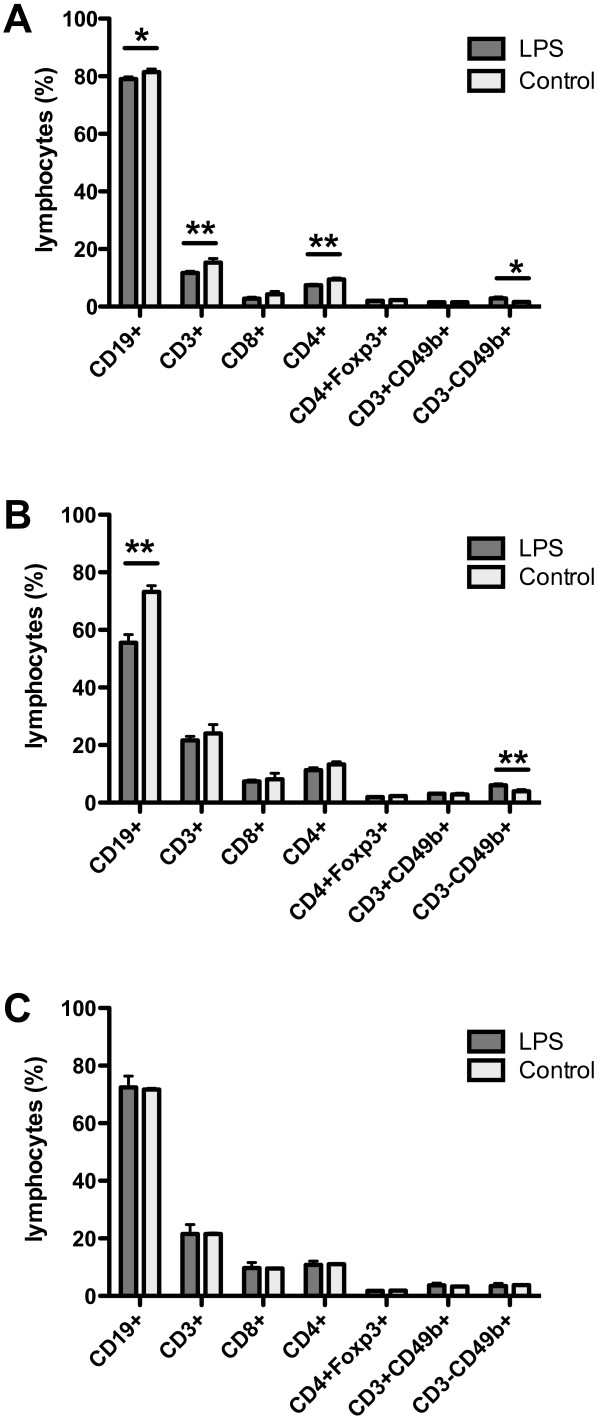
**FACS analysis of lymphocyte subpopulations in the spleens of LPS-treated and control LPS-untreated B10.BR males.** A decreased percentage of B cells (CD19+) and an increased percentage of natural killer cells (CD3-CD49b+) after the second and the fourth LPS dose (**A**, **B**) and a decreased percentage of T cells (CD3+, CD4+) after the second LPS dose (**A**) were found in LPS-treated males. No differences in the percentage of B cells (CD19+), subpopulations of T cells (CD3+, CD8+, CD4+, and CD4+Foxp3+) and natural killer cells (CD3+CD49b+, CD3-CD49b+) were found between both groups 20–22 weeks after the fourth LPS dose (**C**). * p < 0.05, ** p < 0.01.

Significant differences in lymphocyte subpopulations in the spleen were observed between LPS-treated and control LPS-untreated males through the course of LPS administration (Figure 5). FACS analysis of splenocytes demonstrated a decreased percentage of CD19+ B cells (p < 0.05), CD3+ T cells (p < 0.01) and CD4+ T helper cells (p < 0.01) after the second LPS dose (Figure 5A). The decrease in percentage of CD19+ B cells (p < 0.01) persisted and even deepened after the fourth LPS dose (Figure 5B). The percentage of CD8+, CD4+Foxp3+ and CD3+CD49b+ T cells remained unaffected by the LPS treatment (Figure 5A, B). The proportion of CD3-CD49b+ natural killer cells was increased in LPS-treated males compared to control males (p < 0.05 resp. p < 0.01) (Figure 5A, B). At the end of the observation period the expression of B cells (CD19+), T cells (CD3+), cytotoxic T cells (CD8+), helper T cells (CD4+), and regulatory T cells (CD4+Foxp3+) as well as natural killers (CD3-CD49b+) and natural killer T cells (CD3+CD49b+) was comparable and nearly identical in both groups (Figure 5C).

### Cytokine levels in blood sera

**Figure 6:**
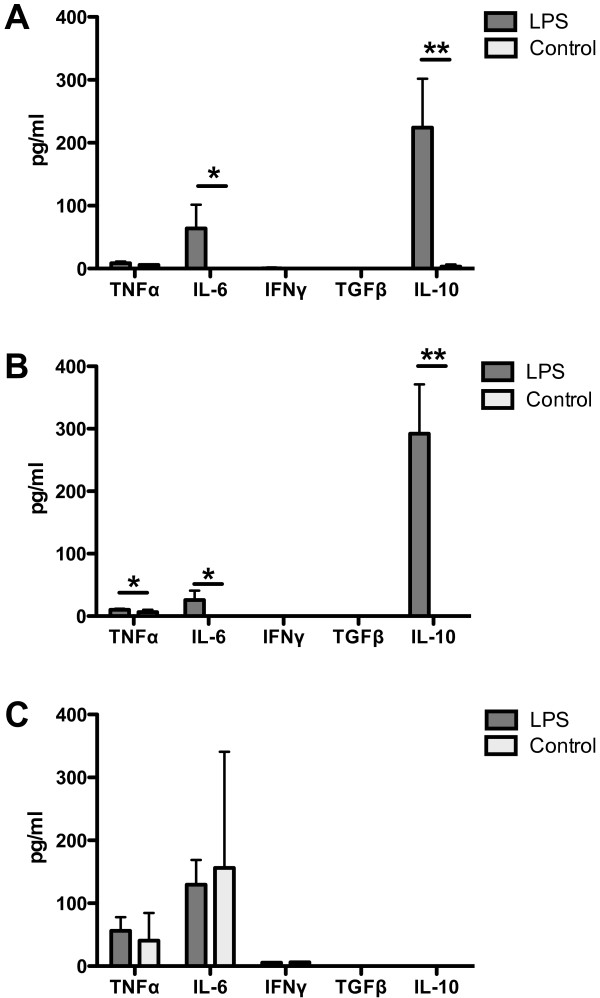
**Cytokine levels in blood sera of LPS-treated and control LPS-untreated B10.BR males.** Values of TNFα, IL-6, IFNγ, TGFβ and IL-10 represent the mean ± SD. IL-6 and IL-10 were significantly increased in LPS-treated males after the second and the fourth LPS dose, TNFα after the fourth LPS dose (**A**, **B**). No significant differences in cytokine levels were found at the end of the experiment, *i.e.* 20–22 weeks after the fourth LPS dose (**C**). TGFβ and in some cases IFNγ and IL-10 were under the limit of the detection kit. * p < 0.05, ** p < 0.01.

The following cytokines were measured: TNFα, IL-6, IFNγ, TGFβ, and IL-10. After the second and the fourth LPS dose there were significantly elevated levels of IL-6 (p < 0.01) and IL-10 (p < 0.01) (Figure 6A, B). At the end of the experiment the levels of TNFα, IL-6, and IFNγ cytokines were not significantly different between the LPS-treated and the control group and the levels of TGFβ and IL-10 cytokines were bellow the detection limit of the method (Figure 6C). However, IL-6 and TNFα were elevated in both LPS-treated and PBS-treated control groups (Figure 6C).

### *In vitro* cytokine response of spleen cells

**Figure 7:**
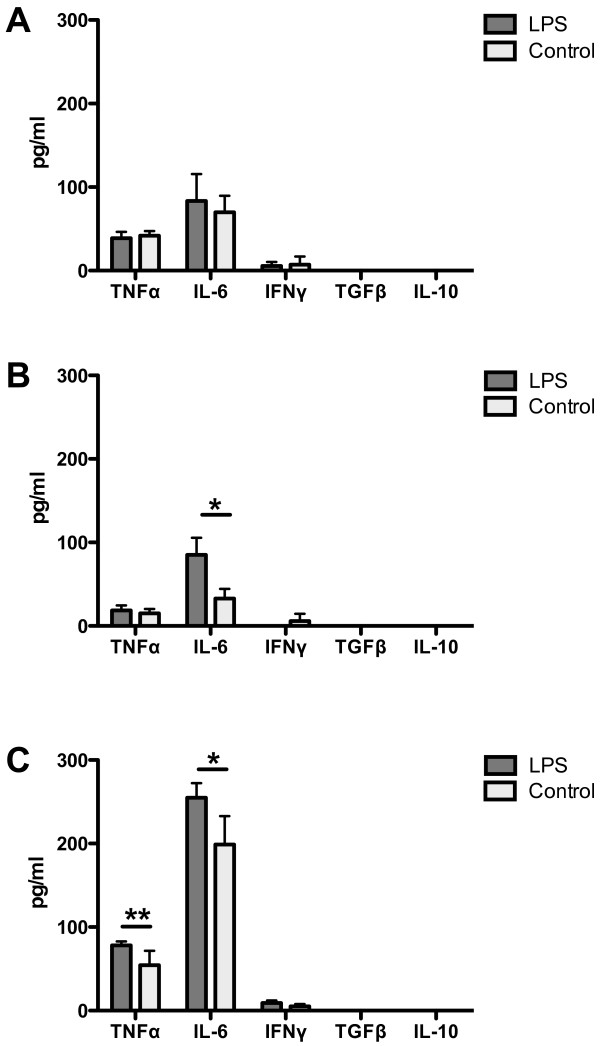
***In vitro* cytokine response of splenocytes from LPS-treated and control LPS-untreated B10.BR males to LPS stimulation.** TNFα, IL-6, IFNγ, TGFβ and IL-10 in cell supernatants were determined. No significant differences were found between LPS-treated males and the control group after the second LPS dose (**A**). After the fourth LPS dose the IL-6 level was significantly increased in LPS-treated males (**B**). At the end of the experiment splenocytes from LPS-treated males responded to LPS stimulation by significantly higher production of TNFα and IL-6 cytokines (**C**). * p < 0.05, ** p < 0.01.

Spleen cells from LPS-treated and control males were stimulated *in vitro* with LPS and the levels of TNFα, IL-6, IFNγ, TGFβ and IL-10 in cell supernatants were evaluated (Figure 7). After the fourth LPS dose the IL-6 level was significantly higher in LPS-treated males (p < 0.05) than in the controls (Figure 7B). Twenty-two weeks after the final LPS dose the splenocytes isolated from LPS-treated males were more susceptible to LPS stimulation and produced significantly higher levels of pro-inflammatory cytokines TNFα (p < 0.01) and IL-6 (p < 0.05) than splenocytes from LPS-untreated control males (Figure 7C).

## Discussion

Bacterial lipopolysaccharide (endotoxin) triggers acute inflammation, associated with activation of the innate immune system and production of multiple proinflammatory mediators [19,20]. In this study, we attempted to utilize the proinflammatory capability of LPS to enhance the incidence of the naturally occurring inflammatory joint disease ANKENT in mice.

ANKENT develops almost exclusively in males and this hallmark of the disease is associated with hormonal and behavioural factors – similarly as in naturally occurring arthritis in DBA/1 male mice [21]. The males caged together have a significantly higher risk of ANKENT development than males caged alone [22]. During the whole experiment both LPS-treated and LPS-untreated males were caged under the same conditions and we have not observed any significant difference in behaviour between the groups.

Previously, we showed that germ-free mice do not suffer from the joint disease [13]. However, after their colonization with a mixture containing common intestinal bacteria, some mice developed ANKENT [14]. The close connection between the gut and SpA was repeatedly demonstrated in laboratory animals [23-27] and was also confirmed in humans [28-33]. Because LPS is an agent which when administered intraperitoneally enhances intestinal permeability, and leads to translocation of commensal microbiota from the gut [17], we expected a higher frequency of the disease in LPS-treated males. Surprisingly, LPS administration to ANKENT-susceptible B10.BR male mice in their early adulthood acted protectively: the incidence of ANKENT in LPS-treated males was significantly reduced compared to control LPS-untreated group. This unexpected result could be explained by an overactivation of innate immunity cells leading to an upregulation of negative regulatory pathways [34].

The second aim of our work was to study the immune response. The cellular infiltrate in joints and elevated IL-6 levels in the sera were demonstrated in ANKENT positive animals at the time when ANKENT became manifest [9,11]. These signs disappeared after the period of acute inflammation. In this study we did not test mice in the acute stages of the disease, but we were interested in the immune response that precedes ANKENT onset. We compared populations of immune cells and cytokine levels in LPS-treated group, where ANKENT occurrence was suppressed, with the control LPS-untreated group, where ANKENT was manifest.

Throughout the course of LPS administration, significantly higher percentages of macrophages (CD11b+), dendritic cells (CD11c+), neutrophils (Ly6G+) and also natural killer cells (CD3-CD49b+) were found in LPS-treated males than in LPS-untreated ones. This LPS-induced expansion of innate immune cells in spleen was transient and was not present at the end of experiment when the frequencies of these populations were similar in both groups.

Just the opposite effect of LPS treatment was observed in lymphocyte subpopulations. During LPS administration the percentage of B cells, T cells and T helper cells in the spleen was significantly decreased in LPS-treated males and the decrease in frequency of B cells lasted throughout LPS treatment. At the end of the experiment, no differences were found between LPS-treated and LPS-untreated males. This suggests that cells of the adaptive immune system did not directly participate in the immune response to LPS treatment, and the suppression of adaptive immunity in LPS-treated males might be involved in the reduction of ANKENT incidence.

Specific cells of innate and adaptive immunity are associated with the production of cytokines. In LPS-treated males we found significantly increased serum levels of IL-6 and IL-10 during LPS administration, whereas the percentage of CD4+ T cells, which is an important source of these cytokines, was decreased. However, macrophages also produce IL-6 and monocytes, whose frequency was strongly elevated in LPS-treated males, are the main cellular source of IL-10.

The finding of significantly elevated IL-10 levels in sera of LPS treated males was discrepant with the *in vitro* response of LPS stimulated splenocytes, which did not secrete detectable IL-10. This contradiction between serum and *in vitro* spleen cell response to LPS stimulation could be explained by the fact that the major source of circulating IL-10 after LPS stimulation is liver [35].

No significant serum differences were found between LPS-treated and LPS-untreated males in IL-6 and IL-10 and in all other tested cytokines at the end of the experiment. However, IL-6 and TNFα were elevated in both groups. The elevation of these pro-inflammatory cytokines at the end of experiment could be explained by the presence of ANKENT-positive animals. We could only speculate that LPS treatment suppressed and delayed the onset of ANKENT and that cytokine data both from serum and from *in vitro* stimulation show the subclinical signs of disease. Nevertheless, at the end of the experiment we have detected only a single affected mouse in LPS-treated group. To evaluate the data in more detail we would need to match single cytokine data with individual mice. However, both LPS-treated as well as LPS-untreated control males were evaluated as a group.

We do not know the exact mechanism of LPS effect in ANKENT development, but the high level of anti-inflammatory cytokine IL-10 could contribute to homeostasis in LPS-treated males. IL-10, which is a potent anti-inflammatory agent, might suppress the onset of ANKENT by its effect on the secretion of pro-inflammatory cytokines including TNFα and IL-6. A regulation of IL-10 is connected with a function of pro-inflammatory TNFα. In mice, TNFα up-regulates LPS-induced IL-10 synthesis and neutralization of TNFα with anti TNFα antibody results in a significant reduction of LPS-inducible IL-10 production [35].

To better understand the immune mechanisms underlying the development of ANKENT*, in vitro* LPS stimulation of splenocytes was performed. In the course of LPS administration we observed an elevated level of IL-6 in supernatants of splenocytes from LPS-treated males. This result was consistent with the elevated IL-6 levels in blood sera from the LPS group. At the end of the experiment we found not only further increase in IL-6 levels but also increase in TNFα levels in both groups. The levels of both TNFα and IL-6 were significantly higher in LPS-treated group compared to control group. TNFα is directly produced by macrophages in response to LPS stimulation [36] and is also a typical marker for AS in man [37]. The anti-TNF monoclonal antibodies that act by neutralizing TNF have proved highly effective in AS therapy, but also in the treatment of other spondyloarthropathies [38,39].

## Conclusions

According to our results we conclude:

1. Repeated systemic stimulation with the microbial component LPS caused an activation of the innate immune system followed by suppression of both arms of the immune system in the LPS-treated males. These changes correlated with the reduction of the ANKENT incidence.

2. Considerable increase in the anti-inflammatory IL-10 level in the LPS-treated males can be a potential mechanism that decreased the ANKENT incidence.

3. Our findings suggest that LPS stimulated development, expansion and function of the immune system so that LPS-treated males were less susceptible to ANKENT development than LPS-untreated controls.

## Abbreviations

ANKENT: Ankylosing enthesopathy; AS: Ankylosing spondylitis; SpA: Spondyloarthropathies; LPS: Lipopolysaccharide; TNFα: Tumor necrosis factor α; IL-6: Interleukin-6; IFNγ: Interferon γ; TGFβ: Transforming growth factor β; IL-10: Interleukin-10; PBS: Phosphate-buffered saline.

## Competing interests

The authors declare that they have no competing interests.

## Authors’ contributions

HTH and JC proposed conception and experimental design. JC, TH and AK carried out LPS treatment, ANKENT screening, immunoassays and acquisition of data. HTH, TH and JC analyzed and interpreted obtained data. JC, HTH and TH drafted the manuscript and revised it critically. All authors read and approved the final manuscript.

## Pre-publication history

The pre-publication history for this paper can be accessed here:

http://www.biomedcentral.com/1471-2474/13/110/prepub
